# Supplementary material for: The Virulence Potential of Livestock-Associated Methicillin-Resistant Staphylococcus aureus Cultured from the Airways of Cystic Fibrosis Patients
Source: Toxins (Basel). 2020 May 30;12(6):360. doi: 10.3390/toxins12060360 (PMC7354617; doi:10.3390/toxins12060360)
Supplement: Supplementary file 1 [file toxins-12-00360-s001.zip › toxins-813122 supplementary materials/toxins-813122 supplementary for proofreading.pdf]

Supplementary Materials: The Virulence Potential of Livestock-Associated Methicillin-Resistant *Staphylococcus aureus* Cultured from the Airways of Cystic Fibrosis Patients

Janina Treffon, Sarah Ann Fotiadis, Sarah van Alen, Karsten Becker and Barbara C. Kahl

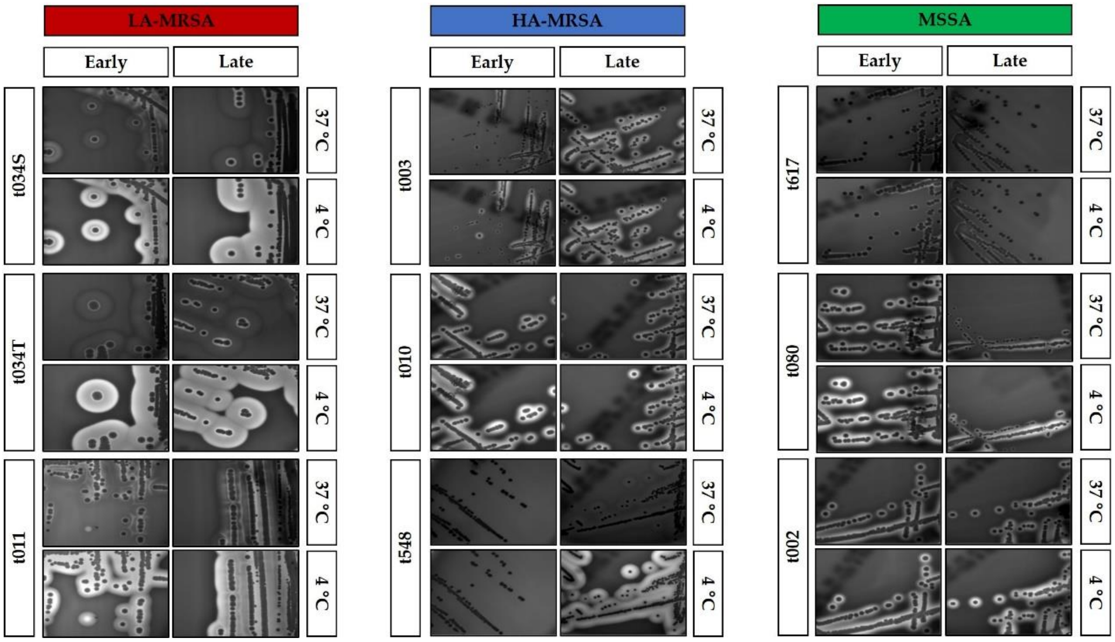

Figure S1. Hemolysis assay on Columbia sheep blood agar. Bacteria were streaked onto Columbia sheep blood agar and incubated at 37 °C and subsequently 4 °C for 24 h, respectively, to determine activity of bacterial hemolysins. Pictures were taken with a digital camera and cropped using IrfanView (Irfan Skiljan, Wiener Neustadt, Austria). Colony sizes of the isolates might differ due to variable camera positions and therefore, cannot be compared among each other. As determination of hemolysis, but not colony size, was focus of the investigation, colony size was not measured. Abbreviations: HA-MRSA (hospital-associated methicillin-resistant *S. aureus*), LA-MRSA (livestock-associated methicillin-resistant *S. aureus*), MSSA (methicillin-susceptible *S. aureus*), S (isolated from sputum), T (isolated from throat).
